# Effect of post-surgical flap position on soft tissue regrowth and keratinized tissue increase following fibre retention osseous resective surgery: a 6-month randomized study with multilevel analysis

**DOI:** 10.1186/s12903-023-03144-2

**Published:** 2023-07-10

**Authors:** Gian Marco Piccoli, Federica Romano, Marta Giraudi, Nicolò La Bruna, Filippo Citterio, Giulia Maria Mariani, Giacomo Baima, Mario Aimetti

**Affiliations:** 1 Department of Surgical Sciences, Section of Periodontology, C.I.R. Dental School, University of Turin, Via Nizza 230, Turin, 10126 Italy; 2 Private Practice, Udine, Italy

**Keywords:** Keratinized tissue, Osseous resective surgery, Periodontitis, Soft tissue rebound

## Abstract

**Background:**

The aim of this randomized split-mouth study was to assess the influence of primary flap position on the amount of coronal soft tissue regrowth and keratinized tissue (KT) 6 months after osseous resective surgery with fiber retention technique (FibReORS).

**Materials and methods:**

Two contralateral posterior sextants in 16 patients were treated with FibReORS and randomly assigned to flap positioning either 2 mm below the bone crest (apical group) or at the level of bone crest (crestal group). Clinical parameters were recorded at 1, 3 and 6 months and patient-related outcomes during the first two post-operative weeks.

**Results:**

Healing period was uneventful. Patient’s discomfort was similar in both groups. The overall soft tissue rebound was higher in the apical than in the crestal group (2.0 ± 1.3 mm *versus* 1.3 ± 0.7 mm), but the difference was statistically significant only interproximally (2.2 ± 1.3 mm *versus* 1.6 ± 0.8 mm). Multilevel analyses showed higher soft tissue rebound in sites with normal compared to thin phenotype (1.5 mm, *p* < 0.0001) and treated with flap positioned 2 mm apically to the bone crest (0.7 mm, *p* < 0.001). An additional 0.5 mm KT increase was observed at interdental sites in the apical group.

**Conclusions:**

Apical flap positioning increases soft tissue rebound and KT width, mainly at the interdental sites, with reduced patient discomfort.

**Trial registration:**

The trial was registered at ClinicalTrials.gov (ID: NCT05140681, Registration date: 1/12/2021, retrospectively registered).

**Supplementary Information:**

The online version contains supplementary material available at 10.1186/s12903-023-03144-2.

## Background

Osseous resective surgery (ORS) is a well-documented treatment approach to eliminate residual periodontal pockets associated with shallow-moderate intrabony defects at posterior teeth [1–4]. This treatment modality resulted in greater probing depth (PD) reduction and lower long-term disease progression than non-surgical therapy and conservative surgery [5, 6]. With the aim of limiting the quantity of resected bone, Carnevale introduced the fibre retention osseous resective surgery (FibReORS), using connective fibres as reference point for the ostectomy [7].

Classical studies have long questioned the possibility of increasing keratinized tissue (KT) following interventions aimed at pocket elimination through the apically repositioned flap (APF). Pontoniero and Carnevale observed a coronal tissue re-growth with a new “physiological” supracrestal gingival unit at 12 months after ORS in periodontally healthy patients [8]. Substantial differences were found depending on the position of the flap after suturing, with a more apical position associated with a greater coronal regrowth [9–11]. However, leaving a consistent part of interproximal and crestal bone exposed after suturing may cause greater post-operative pain. Recently, Cairo et al. compared soft tissue regrowth following ORS and FibReORS techniques in periodontally compromised patients, showing a minimal difference at 12 months favouring ORS [12]. Aimetti et al. observed a comparable coronal soft tissue regrowth at 48 months [13].

To the current state of knowledge, no study has taken into account the re-growth of soft tissue following FibReORS depending on the position of the flap with respect to the bone crest at the end of surgery. In particular, no study examined the amount of KT obtained following healing. This aspect could have a major clinical impact in the long-term maintenance of oral hygiene and periodontal health, especially for teeth in need of prosthetic treatment.

Considering periodontitis patients treated with FibReORS, this study primarily aimed to compare the amount of soft tissue regrowth and KT increase at 6 months according to the position of the primary flap apically or at the level of the bone crest, and secondly to evaluate the patients’ post-operative discomfort.

## Materials and methods

### Study design

This split-mouth, double blind, randomized clinical trial was approved by the Institutional Ethics Committee and was conducted according to the Helsinki Declaration. Informed consent was obtained from each patient. This article was reported according to the CONSORT statement. The study was retrospectively registered at ClinicalTrials.gov (ID: NCT05140681, Registration date: 1/12/2021, retrospectively registered).

Patients were serially recruited from those who had completed the cause-related phase of periodontal therapy at the Section of Periodontology, C.I.R. Dental School, University of Turin. Inclusion criteria were as follows: (1) diagnosis of severe chronic periodontitis [14] (grade III or IV according to the actual classification); (2) good general health; (3) full-mouth plaque score (FMPS) and full-mouth bleeding score (FMBS) < 20%; (4) two contralateral sextants with residual PDs of > 5 mm and persisting bleeding on probing (BoP) at posterior natural teeth 3 months after the completion of cause-related therapy. Exclusion criteria included: (1) pregnancy and lactation; (2) smoking > 10 cigarettes/day; (3) intake of antibiotics in the previous 6 months. Teeth with degree II or III mobility, horizontal bone loss higher than 1/3 of the root length or designed as abutment for prosthetic rehabilitation were also excluded from the study.

Each patient (experimental unit) contributed with two contralateral posterior sextants (surgical area) containing at least 2 pockets/defects with indication to FibReORS. Each sextant was randomly assigned to one of two treatment modalities: FibReORS associated with APF 2 mm below the bone crest (apical group) and FibReORS associated with APF at the level of the bone crest (crestal group). In each sextant, the site associated with the deepest intrabony defect was selected as the experimental site.

### Sample size calculation

The soft tissue regrowth at the experimental site was set as the primary outcome. A sample size of 14 patients was calculated to detect a minimum difference of 1.12 mm between groups with an expected standard deviation of 0.60 mm [8], an alpha error of 5% and a power of 80%. For compensation of possible dropouts, 16 individuals were recruited.

### Randomization and allocation concealment

According to a computer-generated balanced block randomization table with a 1:1 allocation, each of the two sextants in the enrolled patients was assigned to the apical or crestal group. Treatment assignments were concealed in opaque envelopes with the corresponding number on the outside (identifying both patient and sextant). A clinician not involved in the study informed the surgeon after bone remodelling, in which position he should suture the flap. The first sextant was operated 2–3 weeks after the patients’ enrolment, while the contralateral sextant in a time between 6 and 12 weeks after the first surgery.

### Surgical procedures and post-surgical care

Antibiotic (amoxicillin 2 g) and anti-inflammatory therapy (ibuprofen 600 mg) was administered 1 h prior to the surgery. Surgical procedures were performed by one operator using loops 4X under coaxial light as described in detail elsewhere [15]. Bone remodelling was made according to the FibReORS technique to eliminate the infrabony defect and re-shape positive attached fibres/bony architecture [7]. The flap margins were placed at the bone crest level (crestal group) or 2 mm apically (apical group) and secured using vertically external mattress sutures with periosteal anchorage.

Patients avoided brushing in the treated sextants for 2 weeks after surgery and rinsed twice daily for 1 min with a 0.20% chlorhexidine gluconate solution. At week 1, sutures were removed. Patients resumed toothbrushing with soft toothbrush after 2 weeks and interdental cleaning after 4 weeks. Weekly prophylaxis was delivered during the first 3 weeks after surgery and thereafter patients entered a 3-month recall system.

### Clinical outcomes

All measurements were made with a 1-mm graduated periodontal probe (PCP 15, Hu-Friedy, Chicago, IL, USA) by one calibrated and masked clinician. Presence/absence of plaque, presence/absence of BoP and PD were measured at 6 points on the treated teeth at baseline, 3 and 6 months post-surgery. FMPS and FMBS were also recorded.

The distance of the gingival margin (GMD) and the amount of KT were measured using individual acrylic stent, used as fixed-reference mark, at the end of the surgical session, 1 month, 3 and 6 months post-surgery at interdental and buccal aspects [10, 11]. The intra-class correlation coefficients were 0.94 for GMD and 0.86 for KT.

Periodontal phenotype was determined before surgery using a 25 endodontic reamer at the mid-buccal aspect of the experimental tooth, 1 mm apically to the gingival margin level. If there was no attached gingiva, the same measurement was made on the alveolar mucosa [16]. Phenotype was classified as thin if gingival thickness was less than 1 mm, normal if equal to 1 mm, and thick if more than 1 mm [17].

### Surgical and patients-related outcomes (PROs)

Wound healing and patients’ pain/discomfort were assessed at week 1 and 2. Patients scored the intensity using a horizontal visual analogue scale (VAS), 10-cm long (0 = no pain; 10 = extreme pain), one for each treated sextant and one for each follow-up visit [18]. They recorded also the number of painkillers taken and the quality of diet during the first two post-operative weeks using a questionnaire. The diet was classified as normal (solid/soft and hot food), moderately changed (only cold solid/soft food), or severely changed (only cold liquid food) [19].

### Statistical analysis

Statistical analysis was performed using STATA Statistical Software (College Station, TX: StataCorp LP). Repeated measures ANOVA and paired Student t-test with Bonferroni correction were applied for within and between groups analysis, respectively, for quantitative variables at both the surgical area and experimental site and chi-square test for qualitative variables.

Multilevel linear regression models at three levels (Patient, Site and Time) were built to analyse factors associated with KT increase (KT-Inc) and soft tissue rebound (GMD reduction, GMD-Red) at both the surgical area and experimental site. KT-Inc and GMD-Red were calculated as the difference in mm between the corresponding values at 6 months and those at the end of the surgery. At the patient level the covariates were gender, age (years), smoking habit (dichotomous), phenotype (normal/ thick/thin), and treatment (apical/crestal flap). GMD_0_ (baseline GMD in mm) and KT_0_ (baseline KT in mm) were entered at the site level and T1 (1 month), T3 (3 months) and T6 (6 months) at the Time level. Interactions were also estimated. *P* < 0.05 was considered statistically significant.

## Results

### Study population

**Fig. 1:**
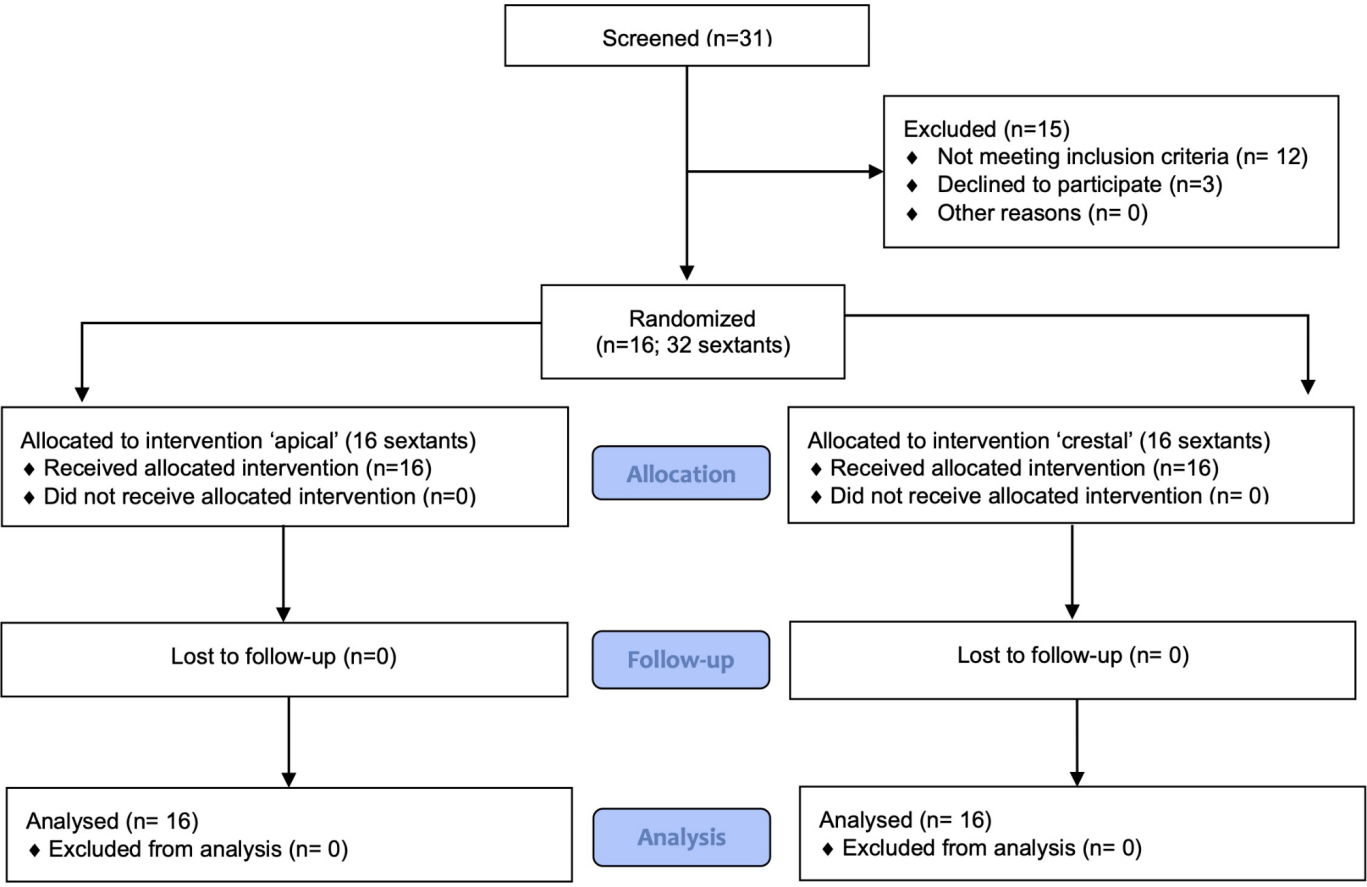
CONSORT flow diagram of patients’ recruitment and follow-up

A total of 31 patients were screened; 12 patients did not meet the inclusion criteria and 3 declined to participate (Fig. 1). Finally, 16 patients (12 females and 4 males, mean age 42.9 ± 9.1 years) were enrolled and completed the trial. Three patients were light smokers. Five patients showed a normal phenotype, five a thin phenotype and six a thick phenotype. All patients completed the trial. The last visit was scheduled in October 2018.

Healing period was uneventful. Interproximal fibrin deposits were detectable in 15 of the 16 treated sextants after both procedures at day 7. At day 14 they were still present in 8 sextants of the apical group and in 5 sextants of the crestal group. Wound healing was completed within 3 weeks.

### Clinical outcomes

FMPS and FMBS remained below 20% throughout the study period. PD values were comparable at baseline between the two procedures. At 6-month examination, shallow PDs were obtained with no differences between the groups (Supplementary Table 1).

**Table 1:** Gingival margin distance (GMD) measured over the experimental period at the surgical area (mean ± SD)

Time	Treatment group								*P-*value		
	Apical				Crestal						
	Overall	Inter-proximal	Buccal		Overall	Inter-proximal	Buccal		Overall	Inter-proximal	Buccal
Baseline	4.3 ± 2.1	3.7 ± 2.0	5.5 ± 2.3		4.6 ± 2.0	4.0 ± 1.7	5.9 ± 2.5		NS	NS	NS
Post-surgery (T0)	8.0 ± 1.9	7.7 ± 1.8	8.5 ± 2.4		7.3 ± 1.7	7.1 ± 1.6	7.8 ± 1.9		NS	NS	NS
1 month (T1)	6.6 ± 1.9	6.2 ± 1.7	7.4 ± 2.4		6.6 ± 2.1	6.2 ± 2.0	7.4 ± 2.4		NS	NS	NS
ΔT0-T1	–1.4 ± 0.9	–1.5 ± 0.9	–1.1 ± 1.2		–0.7 ± 0.6	–0.9 ± 0.6	–0.3 ± 0.7		0.04	0.04	N
3 months (T3)	6.2 ± 2.1	5.8 ± 2.0	7.0 ± 2.4		6.3 ± 2.0	5.8 ± 1.9	7.2 ± 2.3		NS	NS	NS
ΔT0-T_3_	–1.8 ± 1.2	–1.9 ± 1.1	–1.5 ± 1.5		–1.0 ± 0.6	–1.3 ± 0.6	–0.5 ± 0.7		0.04	0.04	0.05
6 months (T6)	6.0 ± 2.0	5.5 ± 1.9	6.9 ± 2.4		6.0 ± 2.1	5.5 ± 2.0	7.0 ± 2.3		NS	NS	NS
ΔT0-T6	–2.0 ± 1.3	–2.2 ± 1.3	–1.6 ± 1.6		–1.3 ± 0.7	–1.6 ± 0.8	–0.8 ± 0.7		NS	0.045	NS

*SD* standard deviation; *NS* not statistically significant

Changes of GMD and KT at the surgical area are presented in Tables 1 and 2. The apical group showed higher GMD than the crestal one at the time of suturing and higher overall tissue regrowth (2.0 ± 1.3 mm versus 1.3 ± 0.7 mm for the apical group, *p* = 0.06). At the interproximal level the coronal displacement was significantly greater in the apical group (2.2 ± 1.3 mm versus 1.6 ± 0.8 mm, *p* = 0.045). Approximately 87.6% and 78.0% of the regrowth occurred during the first 3 months in the apical and crestal groups, respectively. Regarding KT (Table 2), no statistically significant difference was detected at 6 months between the groups, but a statistically significant additional 0.5 mm increase was detected inter-proximally in the apical group (*p* = 0.03).

**Table 2:** Keratinized tissue (mm) measured over the experimental period at the surgical area (mean ± SD)

Time	Treatment group								*P-*value		
	Apical				Crestal						
	Overall	Inter-proximal	Buccal		Overall	Inter-proximal	Buccal		Overall	Inter-proximal	Buccal
Baseline	6.0 ± 1.4	6.6 ± 1.3	4.9 ± 1.6		5.8 ± 1.4	6.4 ± 1.4	4.7 ± 1.7		NS	NS	NS
Post-surgery (T0)	3.3 ± 0.9	3.5 ± 1.0	2.8 ± 0.9		3.6 ± 0.9	3.9 ± 1.0	2.9 ± 1.0		NS	NS	NS
1 month (T1)	4.8 ± 1.1	5.3 ± 1.2	3.9 ± 0.9		4.8 ± 0.9	5.2 ± 0.9	3.9 ± 1.1		NS	NS	NS
ΔT0-T1	1.5 ± 0.9	1.8 ± 1.1	1.0 ± 0.8		1.2 ± 0.8	1.3 ± 0.8	1.0 ± 0.9		NS	NS	NS
3 months (T3)	5.1 ± 1.1	5.7 ± 1.2	4.1 ± 1.0		5.2 ± 0.9	5.7 ± 1.0	4.1 ± 0.9		NS	NS	NS
ΔT0-T_3_	1.9 ± 0.9	2.2 ± 1.0	1.3 ± 0.8		1.6 ± 0.8	1.8 ± 0.9	1.2 ± 0.8		NS	NS	NS
6 months (T6)	5.4 ± 1.1	6.0 ± 1.2	4.2 ± 0.9		5.4 ± 0.9	5.9 ± 0.9	4.2 ± 1.0		NS	NS	NS
ΔT0-T6	2.1 ± 0.9	2.5 ± 1.1	1.4 ± 0.7		1.8 ± 0.9	2.0 ± 1.0	1.3 ± 0.9		NS	NS	NS

*SD* standard deviation; *NS* not statistically significant

**Fig. 2:**
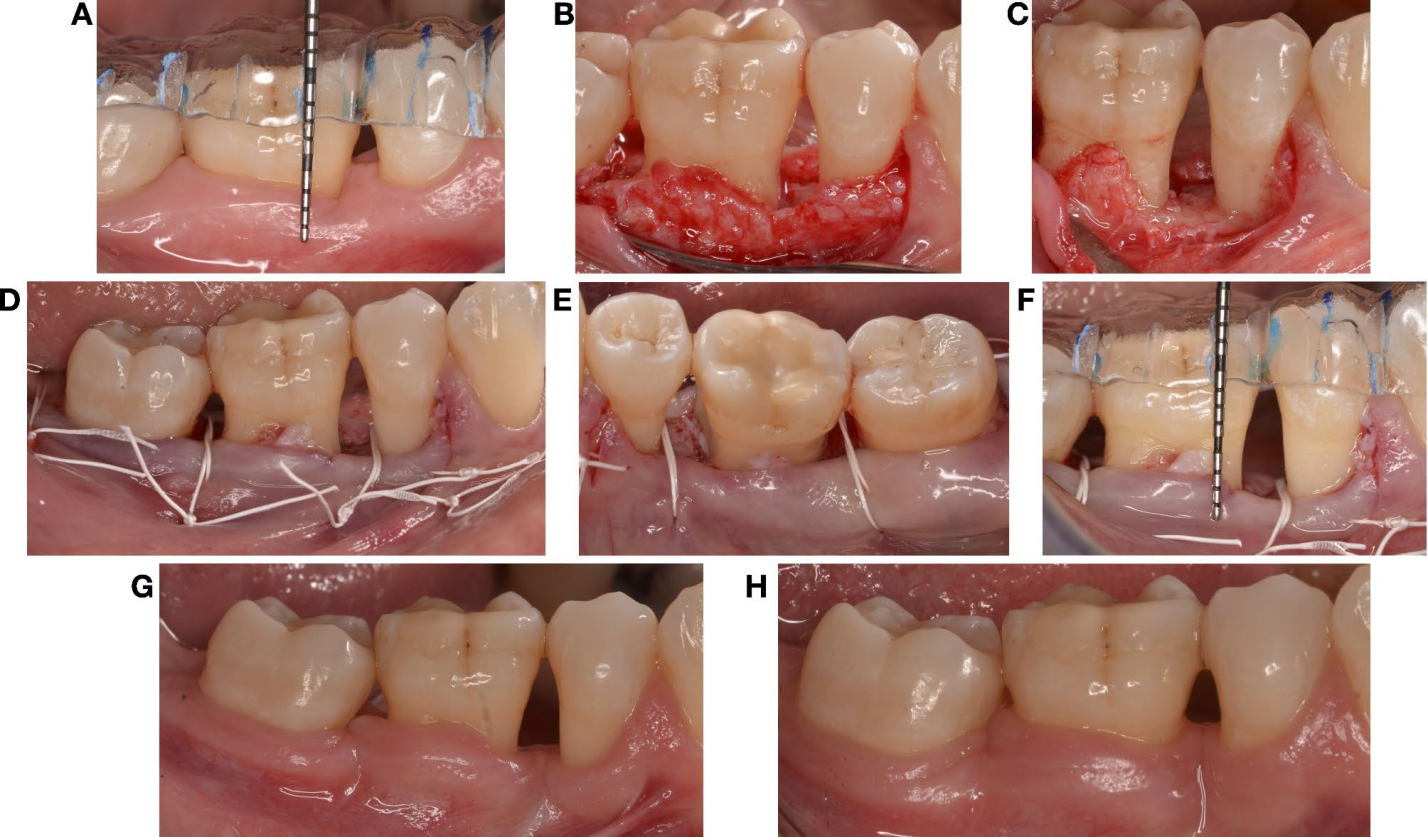
Wound healing and soft tissue regrowth in a sextant treated with FibReORS and apically positioned flap at the level of the bone crest. (**A**) Baseline measurements with individual acrylic stent; (**B**) Intrasurgical view before bone remodelling; (**C**) Intrasurgical view after bone remodelling; (**D**) Sutures (buccal view); (**E**) Sutures (lingual view); (**F**) Post-surgery measurements (**G**) 3 months after surgery; (H) 6 months after surgery

**Fig. 3:**
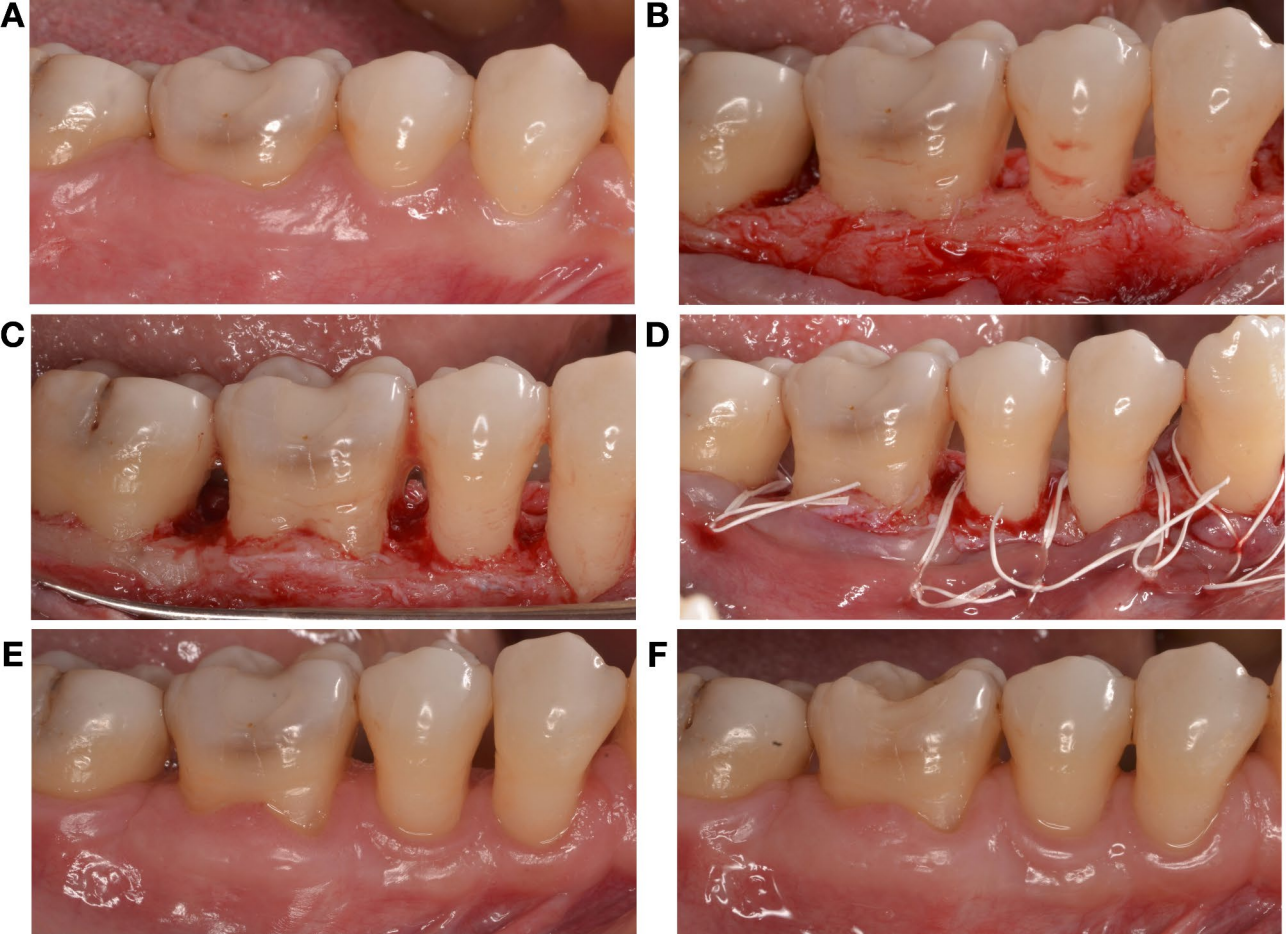
Wound healing and soft tissue regrowth in a sextant treated with FibReORS and apically positioned flap 2 mm below the bone crest. (**A**) Baseline; (**B**) Intrasurgical view before bone remodelling; (**C**) Intrasurgical view after bone remodelling; (**D**) Sutures; (**E**) 3 months after surgery; (**F**) 6 months after surgery

At the experimental site level (Table 3), at 6 months the soft tissue rebound was twice as high in the apical compared to the crestal group (2.6 ± 1.3 mm *versus* 1.2 ± 0.9 mm, *p* = 0.005). Consistently, higher KT increase was detected at both 3- and 6-month examinations in the apical group (*p* = 0.007 and *p* = 0.003). Figures 2 and 3 showed the coronal regrowth in both the experimental groups.

**Table 3:** Gingival margin distance (GMD) and Keratinized tissue width (KT) measured over the experimental period at the experimental site (mean ± SD)

		Time							
Group	Variable	Baseline	Post-surgery (T0)	1 month (T1)	Δ_T0−T1_	3 months (T3)	Δ_T0−T3_	6 months (T6)	Δ_T0−T6_
	**GMD (mm)**								
Apical		3.7 ± 2.4	8.7 ± 2.1	6.7 ± 1.9	–2.0 ± 1.0	6.2 ± 2.1	–2.4 ± 1.0	6.1 ± 2.1	–2.6 ± 1.3
Crestal		5.6 ± 2.7	8.2 ± 23	7.4 ± 2.4	–0.8 ± 0.7	7.1 ± 2.3	–1.1 ± 0.9	6.9 ± 2.2	–1.2 ± 0.9
*P*-value		0.03	NS	NS	0.002	NS	< 0.001	0.18	0.005
	**KT (mm)**								
Apical		6.6 ± 1.8	3.2 ± 1.0	5.2 ± 1.9	2.0 ± 1.5	5.6 ± 1.6	2.4 ± 1.3	6.1 ± 1.7	2.9 ± 1.5
Crestal		5.2 ± 2.5	3.1 ± 1.2	4.1 ± 1.4	1.0 ± 1.0	4.4 ± 1.6	1.3 ± 1.2	4.6 ± 1.6	1.5 ± 1.3
*P*-value		0.07	NS	NS	0.02	0.03	0.007	0.02	0.003

*SD* standard deviation; *NS* not statistically significant

### Multilevel analysis

Factors associated with 6-month GMD-Red are presented in Table 4. At the surgical area, baseline GMD, flap position and time were significantly associated with GMD-Red (*p* < 0.001). The interaction between treatment and time was statistically significant resulting for the coronal group in a lower tissue regrowth of 0.7 mm at 6 months (*p* < 0.001). At the experimental site, higher soft tissue rebound was expected in sites with normal than thin phenotype (1.5 mm, *p* < 0.001).

**Table 4:** Multilevel model to explore factors associated with the 6-month gingival margin distance reduction (mm) at the surgical area and at the experimental site

Term		Surgical Area				Experimental Site			
		Estimate	SE	p-Value	95% CI	Estimate	SE	*p*-value	95% CI
Intercept		3.73	0.89	< 0.001	1.98; 5.47	4.30	1.07	< 0.001	2.19; 6.40
**Patient level**									
Gender		–0.32	0.43	0.46	–1.15; 0.52	–0.57	0.49	0.25	–1.54; 0.39
Age		0.02	0.02	0.28	–0.02; 0.06	0.06	0.02	0.01	0.02; 0.10
Smoke		0.25	0.42	0.56	–0.58;1.07	0.15	0.48	0.75	–0.80; 1.09
Phenotype									
	1	–0.55	0.39	0.15	–1.32; 0.21	–1.46	0.45	< 0.001	–2.34; 0.58
	2	0.19	0.43	0.65	–0.65; 1.04	0.43	0.50	0.39	–0.55; 1.40
**Site level**									
Treatment (0 = Apical)		–0.95	0.25	< 0.001	–1.44; -0.46	–1.60	0.39	< 0.001	–2.35; − 0.83
GMD T0		0.80	0.09	< 0.001	0.62; 0.97	0.60	0.08	< 0.001	0.45; 0.75
**Time level**									
T1		–1.39	0.16	< 0.001	–1.70; -1.08	–2.00	0.20	< 0.001	–2.40; − 1.60
T3		–1.76	0.16	< 0.001	–2.07; -1.45	–2.44	0.20	< 0.001	–2.84; − 2.03
T6		–2.01	0.16	< 0.001	–2.32; -1.70	–2.62	0.20	< 0.001	–3.02; − 2.22
**Interaction**									
Treatment (1 = Crestal)*T1		0.65	0.22	< 0.001	0.21; 1.09	1.19	0.29	< 0.001	0.61; 1.76
Treatment (1 = Crestal)*T3		0.75	0.22	< 0.001	0.31; 1.19	1.31	0.29	< 0.001	0.74; 1.88
Treatment (1 = Crestal)*T6		0.71	0.22	< 0.001	0.27; 1.15	1.38	0.29	< 0.001	0.80; 1.94
**Variances**									
σ^2^n patient		0.12	0.15			1.94^− 11^			
σ^2^u site		0.29	0.13			0.71	0.19		
σ^2^e time		0.20	0.02						

Theoretic model at the surgical area and at the experimental site: GMDijk = β0ijk + β1k Gender (0 = female) + β2k Age + β3k Smoke (0 = No) + β4k Phenotype (0 = thin) + β5jk Treatment (0 = Apical) + β6jk GMD T0 + β7ijk T1 (GMD 1 month respect to GMD at surgery) + β8ijk T3 (GMD 3 months respect to GMD at surgery) + β9ijk T6 (GMD 6 months respect to GMD at surgery) + β10ijk Treatment (0 = Apical)*T1 + β11ijk Treatment (0 = Apical)*T3 + β12ijk Treatment (0 = Apical)*T6.

σ2n, σ2u, and σ2e indicate the variances at the Patient, at the Site and at the Time level respectively. In the theoretic model formula, the subscript k refers to the Patient level. The subscript j refers to the Site level. The subscript i refers to the Time level. b0ijk is the “intercept”. *SE* standard error; *Apical* Flap apical to the bone crest; *Crestal* Flap at the level of the bone crest

Factors associated with 6-month KT-Inc are presented in Table 5. At the surgical area smoking (*p* = 0.04), baseline KT values (*p* < 0.001) and time (*p* < 0.001) were significantly associated with KT-Inc. There was no interaction between time and treatment. At the experimental site, the interaction between treatment and time was statistically significant favoring the apical group (*p* ≤ 0.01), with an additional KT increase of 0.9 mm at 6 months (*p* = 0.015).

**Table 5:** Multilevel model to explore factors associated with the 6-month keratinized tissue increase (mm) at the surgical area and at the experimental site

Term		Surgical Area				Experimental Site			
		Estimate	SE	p-Value	95% CI	Estimate	SE	*p*-value	95% CI
Intercept		1.60	0.94	0.08	–0.24; 3.44	0.28	1.03	0.78	–1.73; 2.30
**Patient level**									
Gender		–0.54	0.31	0.08	–1.14; 0.06	–0.43	0.42	0.30	–1.25; 0.39
Age		–0.02	0.01	0.09	–0.05; 0.00	–0.00	0.02	0.82	–0.04; 0.03
Smoke		0.66	0.32	0.04	0.04;1.28	0.42	0.43	0.32	–0.41;1.26
Phenotype									
	1	0.34	0.29	0.24	–0.22; 0.90	0.32	0.36	0.37	–0.38; 1.03
	2	–0.09	0.29	0.75	–0.66; 0.47	–0.07	0.39	0.85	–0.85; 0.70
**Site level**									
Treatment (0 = Apical)		0.22	0.19	0.23	-0.15; 0.59	0.48	0.37	0.19	–0.24; 1.20
KT T0		0.44	0.09	< 0.001	0.26; 0.63	0.46	0.07	0.001	0.32; 0.60
**Time level**									
T1		1.38	0.11	< 0.001	1.17; 1.60	2.00	0.26	< 0.001	1.48; 2.52
T3		1.75	0.11	< 0.001	1.54; 1.97	2.44	0.26	< 0.001	1.92; 2.96
T6		1.97	0.11	< 0.001	1.76; 2.19	2.87	0.26	< 0.001	2.35; 3.40
**Interaction**									
Treatment (1 = Crestal)*T1		/	/	/	/	–1.00	0.37	0.01	–1.73; − 0.26
Treatment (1 = Crestal)*T3		/	/	/	/	–1.12	0.37	< 0.001	–1.86; − 0.39
Treatment(1 = Crestal)*T6		/	/	/	/	–1.37	0.37	< 0.001	–2.11; 0.64
**Variances**									
σ^2^n patient		1.54^− 14^				4.08^− 7^			
σ^2^u site		0.26	0.07			0.45	0.13		
σ^2^e time		0.19	0.02			0.56	0.06		

Theoretic model at the surgical area: KT ijk = β0ijk + β1k Gender (0 = female) + β2k Age + β3k Smoke (0 = No) + β4k Phenotype (0 = thin) + β5jk Treatment (0 = Apical) + β6jk KT T0 + β7ijk T1 (KT 1 month respect to KT at surgery) + β8ijk T3 (KT 3 months respect to KT at surgery) + β9ijk T7 (KT 6 months respect to KT at surgery)

Theoretic model at the experimental site: KT ijk = β0ijk + β1k Gender (0 = female) + β2k Age + β3k Smoke (0 = No) + β4k Phenotype (0 = thin) + β5jk Treatment (0 = Apical) + β6jk KT T0 + β7ijk T1 (KT 1 month respect to KT at surgery) + β8ijk T3 (KT 3 months respect to KT at surgery) + β9ijk T6 (KT 6 months respect to KT at surgery) + β10ijk Treatment (0 = Apical)*T1 + β11ijk Treatment (0 = Apical)*T3 + β12ijk Treatment (0 = Apical)*T6.

σ2n, σ2u, and σ2e indicate the variances at the Patient, at the Site and at the Time level respectively. In the theoretic model formula, the subscript k refers to the Patient level. The subscript j refers to the Site level. The subscript i refers to the Time level. β0ijk is the “intercept”. *SE* standard error; *Apical* Flap apical to the bone crest; *Crestal* Flap at the level of the bone crest

### PROs

**Fig. 4:**
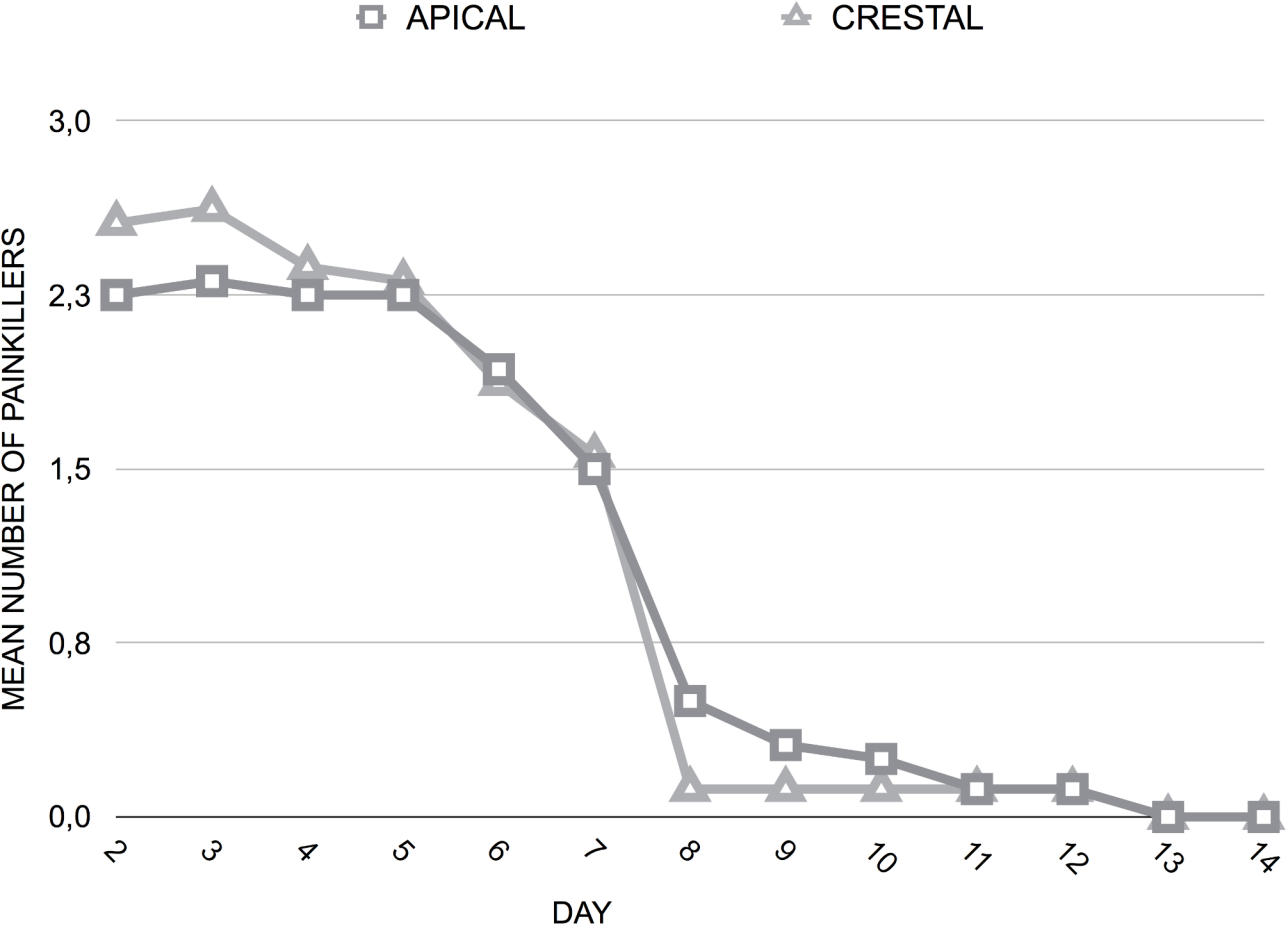
Painkillers consumption at different postoperative times according to postoperative position of the gingival margin

The extent of discomfort/pain, the changes in the dietary habits (Supplementary Table 2), as well as the consumption of analgesic medication (Fig. 4) were comparable between the two procedures during the first two post-therapy weeks.

## Discussion

The present RCT demonstrated a higher coronal soft tissue rebound and KT increase over 6-month healing period following FibReORS when APF was placed 2 mm apically to the bone crest instead at the bone level. A split-mouth design was used to reduce biological variability.

While several studies analysed soft-tissue behaviour following crown lengthening in patients with healthy periodontium [8–11, 20–22], scanty data are available following ORS and FibReORS in periodontitis patients. In particular, no study investigated the influence of flap position on the healing outcomes.

We applied the FibReORS technique because it has been shown similarly effective as ORS for PD reduction with less extent of bone removal and faster soft tissue healing [23]. The present findings confirmed its efficacy in achieving and maintaining minimal PDs over a 6-month period [15, 23].

The marginal periodontal tissue showed a tendency to grow in a coronal direction from the level defined after flap suture in both groups over the 6 months, with a mean GMD-Red of 2.0 mm for the apical group and 1.3 mm for the crestal group, suggesting a tendency of the periodontium to reform a new “physiological” supracrestal gingival unit [8, 13]. When considering only the interdental sites the differences between them were even more pronounced (2.3 mm *versus* 1.6 mm), reaching statistical significance. These findings are consistent with those by Cairo et al. [12], reporting an overall mean GMD-Red of 2.0 mm in sextants treated with FibReORS and APF at the bone crest level, which increased to 2.2 mm inter-proximally. However, the authors considered CEJ as the reference point to measure the soft-tissue coronal rebound, while we used a customized acrylic stent to enhance measurement reliability.

Similarly, Aimetti et al. [15] observed an overall coronal re-growth of 1.6 mm in the surgical area treated with FibReORS and of 3.0 mm at the experimental sites, identified as those with greater baseline PD, at 12 months. In the present study, the amount of soft tissue re-growth at the experimental site level was twice as high in the apical than in the crestal group (2.6 mm v*ersus* 1.2 mm), but data are referred to 6-month behaviour. As previously reported, the periodontium tends to recreate a supracrestal gingival unit re-establishing a supracrestal connective attachment [24]. We observed that soft-tissue rebound had already started after 1 month. At the 3-month follow-up, 87% of coronal rebound had occurred for the apical group compared to 78% for the crestal one. It should be taken into account that a strict monitoring of plaque accumulation was performed, which is critical for a physiological soft-tissue healing process to occur [25].

The role of KT width in the maintenance of periodontal health has been debated for many years and remains controversial. Several site-related conditions, such as gingival recessions, thin phenotype, and root prominence, combined with reduced or missing amount of attached gingiva, may impair maintenance of proper oral hygiene and periodontal health and, thus, may be indication to perform augmentation procedures [26, 27]. Agudio et al. confirmed findings from previous studies about the beneficial contribution of the attached gingiva on periodontal stability [28, 29]. In the current study higher KT gain was obtained in the apical compared to the crestal group at 6 months at both the surgical area (2.1 mm *versus* 1.8 mm) and the experimental site level (2.9 mm *versus* 1.5 mm).

Differences in the amount of soft tissue regrowth and KT increase could be related to specific biological mechanisms triggered during the healing process [30]. Histologically, periosteal retention limits the amount of both bone resorption and granulation tissue production leading to a thinner connective tissue layer formation and to a faster wound re-epithelialization as compared to the complete bone denudation. In this study, flap positioning 2 mm below the bone crest may have stimulated granulation tissue proliferation from bone marrow space and periodontal ligament with consequent formation of higher band of KT and greater tissue rebound [31].

Factors associated with both soft tissue re-growth and KT changes were also investigated using multilevel models. The estimated difference in KT gain between apical and crestal groups was constant over time and amounted to 0.2 mm favouring the apical group. Instead, considering the experimental site, there was a positive interaction between treatment and time of observation. At 6 months the apical group achieved a 0.9 mm higher KT increase than the crestal group. Regarding the soft tissue re-growth, the multilevel model showed a positive interaction between treatment and time of observation with 0.7 mm lower soft tissue rebound at the surgical area for the coronal flap at 6 months post-surgery.

Thick periodontal tissue has been associated with higher soft-tissue rebound after crown lengthening procedures [8]. In this study, normal gingival phenotype was associated with greater tendency to coronal displacement than thin phenotype at the experimental site. This discrepancy may be related to differences in definitions of periodontal phenotype, in reference points used to measure soft-tissue re-growth and in the healing response between patients with periodontitis and healthy periodontium. Further studies are warranted to investigate this aspect. Also, smoking increased the KT width at 6 months even though the three smoker patients daily consumed less than 10 cigarettes.

It is noteworthy that there were no differences in the quality of wound healing and in the intensity of postoperative discomfort. The re-epithelisation was completed after 3 weeks for both groups. Pain-related VAS values as well as the mean quantity of analgesic tablets taken during the first two weeks after surgery were similar between the two groups.

### Limitations

One limitation of this study may be related to the length of follow-up set at 6 months. Nevertheless, the most relevant proportion of soft tissue re-growth occurs during this interval and only minor changes are to be expected thereafter [12, 13]. Secondly, flap repositioning may be regarded as an operator dependent endpoint especially in relation to the need of suturing it 2 mm below the crest. However, an acrylic stent was employed to standardize the reference points and to increase the reproducibility of measurements. Finally, we enrolled into the study only non- or light smokers. This may limit the generalizability of the present results. While smoking is not an absolute contraindication to ORS, it is expected that heavy smokers experience less favourable healing following surgery as well as an increased risk of relapse during post-surgical maintenance [32, 33].

## Conclusions

This study confirmed the benefits of FibReORS in the treatment of shallow-moderate intrabony defects and demonstrated that the placement of the primary flap apically to the bone crest resulted in greater soft tissue regrowth and formation of wider band of KT without any impact on the healing process and patient discomfort. This trend appeared statistically significant at the interdental level and at the experimental sites associated with the greatest intrabony defects.

### Electronic supplementary material

Below is the link to the electronic supplementary material.

Supplementary Material 1: **Supplementary Table 1** Probing Depth (mm) over the experimental period at the surgical area (mean ± SD). **Supplementary Table 2** Patients reported outcomes.

## Data Availability

The datasets used and/or analyzed during the current study are available from the corresponding author on reasonable request.

## List of abbreviations

APF: Apically repositioned flap
BoP: Bleeding on probing
FibReORS: Osseous resective surgery with fiber retention technique
FMBS: Full-mouth bleeding score
FMPS: Full-mouth plaque score
GMD: Distance of the gingival margin
KT: Keratinized tissue
ORS: Osseous resective surgery
PD: Probing depth
PRO: Patient reported outcome
RCT: Randomized clinical trial
VAS: Horizontal visual analogue scale
