# Supplementary material for: Effect of post-surgical flap position on soft tissue regrowth and keratinized tissue increase following fibre retention osseous resective surgery: a 6-month randomized study with multilevel analysis
Source: BMC Oral Health. 2023 Jul 10;23:472. doi: 10.1186/s12903-023-03144-2 (PMC10332078; doi:10.1186/s12903-023-03144-2)
Supplement: Supplementary file 1 — Supplementary Material 1: Supplementary Table 1 Probing Depth (mm) over the experimental period at the surgical area (mean ± SD). Supplementary Table 2 Patients reported outcomes. [file 12903_2023_3144_MOESM1_ESM.docx]

**Supplementary Tables**

**Supplementary Table 1** Probing Depth (mm) over the experimental period at the surgical area (mean ± SD)

|  | **Treatment group** | | | | | | |  |  | | |
| --- | --- | --- | --- | --- | --- | --- | --- | --- | --- | --- | --- |
| **Time** | **Apical** | | |  | **Crestal** | | |  | ***P*-value** | | |
|  | Overall | Inter-proximal | Buccal/  Lingual |  | Overall | Inter-proximal | Buccal/  Lingual |  | Overall | Inter-proximal | Buccal/  Lingual |
| **Baseline** | 4.2 ± 0.39 | 4.9 ± 0.3 | 2.8 ± 0.9 |  | 4.3 ± 0.4 | 5.1 ± 0.5 | 2.7 ± 0.7 |  | NS | NS | NS |
| **1 month (T1)** | 1.2 ± 0.2 | 1.2 ± 0.2 | 1.1 ± 0.2 |  | 1.1 ± 0.1 | 1.2 ± 0.2 | 1.0 ± 0.1 |  | NS | NS | NS |
| **3 months (T3)** | 1.4 ± 0.1 | 1.5 ± 0.2 | 1.1 ± 0.2 |  | 1.4 ± 0.2 | 1.6 ± 0.2 | 1.1 ± 0.2 |  | NS | NS | NS |
| **6 months (T6)** | 1.7 ± 0.2 | 2.0 ± 0.2 | 1.1 ± 0.3 |  | 1.7 ± 0.2 | 2.0 ± 0.2 | 1.1 ± 0.3 |  | NS | NS | NS |

*SD* standard deviation.

**Supplementary Table 2** Patients reported outcomes

| **Variable** | **Treatment group** | | ***p*-value** |
| --- | --- | --- | --- |
|  | Apical (n=16) | Crestal (n=16) |  |
| **Pain (VAS units)** |  |  |  |
| Day 7 | 4.41 ± 2.65 | 3.69 ± 3.03 | NS |
| Day 14 | 2.53 ± 1.86 | 1.72 ± 2.63 | NS |
| **Diet (n, [%])** |  |  |  |
| Day 7 |  |  | 0.51 |
| *Normal diet* | 3 (18.75%) | 2 (18.75%) |  |
| *Moderate changes in diet* | 9 (56.25%) | 8 (56.25%) |  |
| *Severe changes in diet* | 4 (25%) | 3 (25%) |  |
| Day 14 |  |  | NS |
| Normal diet | 9 (56.25%) | 10 (62.50%) |  |
| Moderate changes in diet | 7 (43.75%) | 6 (37.50%) |  |
| Severe changes in diet | 0 (0%) | 0 (0%) |  |

*VAS* Visual Analogue Scale.
